# *Agrobacterium*-Mediated Gene Transfer to Cereal Crop Plants: Current Protocols for Barley, Wheat, Triticale, and Maize

**DOI:** 10.1155/2009/835608

**Published:** 2009-06-21

**Authors:** Goetz Hensel, Christine Kastner, Sylwia Oleszczuk, Jan Riechen, Jochen Kumlehn

**Affiliations:** ^1^Plant Reproductive Biology, Leibniz Institute of Plant Genetics and Crop Plant Research (IPK), Corrensstraße 3, 06466 Gatersleben, Germany; ^2^Plant Breeding and Acclimatization Institute, Radzików, 05-870 Blonie, Poland

## Abstract

The development of powerful “omics” technologies has enabled researchers to identify many genes of interest for which comprehensive functional analyses are highly desirable. However, the production of lines which ectopically express recombinant genes, or those in which endogenous genes are knocked down via stable transformation, remains a major bottleneck for the association between genetics and gene function in monocotyledonous crops. Methods of effective DNA transfer into regenerable cells of immature embryos from cereals by means of *Agrobacterium tumefaciens* have been modified in a stepwise manner. The effect of particular improvement measures has often not been significantly evident, whereas their combined implementation has resulted in meaningful advances. Here, we provide updated protocols for the *Agrobacterium*-mediated generation of stably transgenic barley, wheat, triticale and maize. Based upon these methods, several hundred independent transgenic lines have been delivered, with efficiencies of inoculated embryos leading to stably transgenic plants reaching 86% in barley, 10% in wheat, 4% in triticale, and 24% in maize.

## 1. Introduction

Cereals such as barley, wheat, triticale and maize play a pivotal role for the nutritional intake of humans, being such via direct utilisation as food or through livestock breeding. For 2007, the FAO estimated a global production of some 700 Mt of maize, over 600 Mt of wheat, 137 Mt of barley and 13 Mt of triticale [1]. This data may explain why these crops are a focus of research and biotechnological development. 

Over the past centuries improvement of cereals was achieved mostly by conventional breeding. However, due to the ever-growing world population, limited availability of water, increasingly exhausted fossil energy resources, and the changing climatic conditions, new technologies are urgently required to cope with future challenges. Since the mid 1990s, genetic engineering of cereals has provided a novel field of opportunities for faster and more directed modification or introduction of agronomically useful traits [2–6]. While the first successful genetic transformation events in cereal species had been based on direct gene transfer, which was associated with a number of disadvantages, the pioneering study of Hiei et al. [7] on *Agrobacterium*-mediated transformation of rice represents another milestone. They generated many independent transgenic plants, with T-DNA being stably integrated in the nuclear genome, and the transgenes were shown to be expressed. Ishida and colleagues [8] were then the first to publish a protocol for the generation of transgenic maize, which also relied on *A. tumefaciens*. In the following years, similar protocols for all major cereal crops including barley [9] and wheat [10] were published.

The ability to efficiently form shoots originating from single totipotent cells is indispensible for successful genetic transformation of plants. In contrast to dicotyledonous plants, cereal crops are hardly able to regenerate plants from leaf tissue. However, other gene transfer target explants, for example, immature embryos [4], embryogenic pollen cultures [11] and isolated ovules [12] have proven useful in cereals. *Agrobacterium*-mediated genetic transformation of cereals has been largely confined to particular genotypes that combine the amenability to gene transfer by *Agrobacterium* with adequate regeneration potential. Besides the most suitable lines used as models in routine transformation, namely, the cv. “Golden Promise” in barley [9], Hi II hybrids in maize [4, 13], and the breeding line “Bobwhite 26” in wheat [10], some other genotypes have turned out to be useful for *Agrobacterium*-mediated transformation, albeit with significantly lower efficiency [3, 5, 14–18]. In triticale, the winter type cv. “Bogo” was shown to perform exceedingly well in plant regeneration from immature embryo explants [19–21]. However, no study has yet provided ample evidence of genomic integration of recombinant DNA by means of *A. tumefaciens* in this cereal crop species.

It is not surprising that protocols efficiently used for cereal transformation generally rely on the use of hypervirulent *Agrobacterium* strains such as EHA101 and EHA105 in maize [22], AGL-0 and AGL-1 in barley and wheat [9, 18, 23–26] as well as hypervirulent derivatives of LBA4404 in maize, barley and wheat [11, 18, 27–29]. Hypervirulence can be mediated by accessory *Vir* genes that are either contained on particular Ti-plasmids [30], on so-called superbinary vectors [31], or on an additional plasmid present in the *Agrobacterium* clone employed [11]. 

Particular attention has to be paid to the binary vectors used for cereal transformation. Many binary vectors that had been developed for dicot species turned out not to be suitable for cereals, which is mainly due to inappropriate promoters and selectable marker genes. Moreover, an exceedingly high stability of the plasmids in *Agrobacterium* appears to be vital so as to provide an adequate proportion of transformation-competent bacteria throughout the entire episode of co-cultivation, in which there are no selective conditions in terms of the bacterial resistance mediated by the binary vector. In this regard, pVS1-based vector backbones proved particularly valuable [32]. More recently, the IPKb vector series was developed that features a number of useful plasmid elements such as pVS1, monocot-compatible promoters and selectable marker genes combined with GATEWAY-cassettes for either over-expression or RNAi-constructs. Moreover, convenient modularity is provided in terms of the selectable marker expression unit and the promoter that directs candidate gene expression [33]. 

A successful interaction of *A. tumefaciens* with the gene transfer recipient cells depends on many particular conditions. In cereals, which are at best untypical *Agrobacterium* hosts, deviations from optimal conditions are hardly tolerated. Influencing variables which are thought to be most crucial for gene transfer events to occur during co-cultivation include nutrient concentrations, temperature, pH, presence and concentration of *Acetosyringone* and antioxidants as well as duration. 

Here, we present updated *Agrobacterium*-based transformation protocols for barley, wheat, triticale and maize, which have been developed and successfully employed to produce hundreds of independent transgenic lines.

## 2. Materials

### 2.1. A. tumefaciens Strains

Transformation of *barley*, *wheat*, and *triticale* was mediated by a hypervirulent derivative of *A. tumefaciens* strain LBA4404 [34] harbouring the binary vector pSB187 that contains the *Hpt* selectable marker gene driven by a 400 bp *CaMV35S*-promoter, the *sgfp* (S65T) reporter gene [35] driven by the maize *Ubi-1*-promoter [36] and the vector backbone from pLH vectors [37] with its borders derived from a nopaline Ti plasmid. 

In *maize*, gene transfer was conducted with the *A. tumefaciens* strain EHA105 [22] containing the binary vector pGH218 with the *Pat* gene as selectable marker and a *Gus*-intron reporter gene under the control of a doubled enhanced *CaMV35S*-promoter [38]. The vector backbone of pGH218 is the same as in pSB187.

The vector plasmids were introduced into *Agrobacterium* by electroporation.

### 2.2. Growth of Donor Plants

Germination of *barley* (*Hordeum vulgare* L.) spring type cv. “Golden Promise”, *wheat* (*Triticum aestivum* L.) winter type cv. “Certo” and *triticale* (*x Triticosecale* Wittmack) winter type cv. “Bogo” grains was conducted in trays filled with a substrate mix (Spezialmischung Petuniensubstrat, Klasmann, Germany) (see Note 1) in a growth chamber (14/12°C day/night, 12 hours light, 136 *μ*mol s^−1^ m^−2^ photon flux density). After 3 weeks small plantlets were either incubated for additional eight weeks in a vernalisation chamber with 4°C and 8 hours light per day, or transferred into 18-cm pots (2.5 L). At the beginning of tillering stage 15 g Osmocote (Scotts, Netherlands) was applied per pot. Further fertilization was conducted by watering the plants fortnightly with 0.3% Hakaphos Blau (Compo, Germany). When the stems started to elongate the plants were transferred to a controlled glasshouse (18/16°C day/night, 16 hours light, and 170 *μ*mol s^−1^ m^−2^ photon flux density). There, they were fertilized only once with 0.3% Hakaphos Grün (Compo, Germany) when the heading commenced (see Note 2). 

*Maize* (*Zea mays* L.) line “Hi II” grains were grown in 9-cm pots (0.25 L) containing a cultivation substrate (Substrat 2, Klasmann, Germany) in a growth chamber (22/20°C day/night, 13 hours light, 170 *μ*mol s^−1^ m^−2^ photon flux density). Three weeks later plantlets were transferred to a controlled glasshouse cabin (25/17°C day/night, 16 hours light, 170 *μ*mol s^−1^ m^−2^ photon flux density) in 35-cm pots (20 L) with a substrate mix and 60 g Osmocote Pro (Scotts, Netherlands) per pot for fertilization (see Notes 3, 4).

### 2.3. Plant Tissue Culture Media

The nutrient media used are summarised in Table 1. According to the protocols, precultivation media (PCM), pretreatment medium (PTM), infection medium (IM), liquid or solid co-culture media (CCM) as well as solid media for callus induction (CIM) and regeneration (RM) are required. PCM, PTM, CCM and CIM used in *barley*, *wheat* and *triticale* are based on MS mineral salts [39] supplemented with additional components as shown in Table 1. The RM medium is based on K4N medium which was published elsewhere [11]. In *maize* IM, CCM and CIM are based on Chu N6 mineral salts [40], and RM is based on MS mineral salts [39] supplemented with additional components as shown in Table 1. The pH was adjusted prior to filter sterilisation of the solutions. For the preparation of solid media, one volume of fourfold concentrated solution was mixed with three volumes of adequately concentrated Phytagel (Sigma, Germany) that had been autoclaved with the respective proportion of distilled water. If not stated otherwise standard 9-cm petri dishes (Greiner, Germany) were used.

### 2.4. Isolation of Immature Embryos and Co-cultivation with A. tumefaciens

For the isolation of immature embryos (IEs) and their subsequent co-cultivation with *A. tumefaciens*, the following materials are needed.

1. Forceps, scalpel, spatula, and preparation needles.
2. Preparation microscope.
3. 6-well cell culture plates (Greiner, Germany).
4. Petri dishes (ø 5.5 cm, Greiner, Germany).
5. Pipettes and disposable tips (200–1000 *μ*L and 1000–5000 *μ*L, autoclaved).
6. Eppendorf tubes (2 mL, autoclaved, Eppendorf, Germany).
7. Filter paper (several sizes, autoclaved, Millipore, Germany).
8. Exsiccator and vacuum pump.
9. Magnetic stirrer.

## 3. Procedures

### 3.1. Isolation of Immature Embryos

In *barley*, *wheat*, and *triticale*, developing caryopses were harvested 12–16 d post pollination, immersed for 3 minutes in 70% ethanol, incubated in 5% sodium hypochlorite supplemented with 0.1% Tween for 15 minutes and washed five times in sterile, distilled water. 

*Barley* IEs were excised from the caryopses by using forceps and a lanzet needle (see Note 5). The embryo axes of the IEs were removed. The IEs were transferred into 2.5 mL liquid BCCM (Table 1) in a 6-well plate with up to 50 IEs per well (see Notes 6–8). 

*Triticale* and *wheat* IEs were excised as described for *barley*, yet without removal of the embryo axes. Fifty IEs were placed per petri dish with the scutellum facing up on TPCM or WPCM, respectively (Table 1). 

*Maize* ears were harvested 10–14 d after pollination when IEs were of 1.5–2.5 mm in length. For surface sterilization the ears were first incubated 5 minutes in 70% ethanol, then in 2.4% sodium hypochlorite supplemented with 0.1% Tween for 20 minutes and finally washed 4 times in sterile distilled water for 5 minutes each. After removing the abaxial top of the kernels with a scalpel, IEs were dissected with a lancet and up to 200 collected in a 2-mL tube containing 1 mL IM (Table 1).

### 3.2. Growth of Agrobacterium and Co-cultivation of Immature Embryos

*A. tumefaciens* strain LBA4404 was grown in 10 mL of antibiotic-free CPY medium [34] overnight at 28°C in 100-mL Erlenmeyer flasks with shaking at 180 rpm (see Note 9). A glycerol stock (200 *μ*L from a growing culture with an OD_600_ of 2.0 and 200 *μ*L of 15% glycerol) stored at − 80°C was thawed and added to the medium so as to start the culture. In case of *maize* transformation, CPY medium was solidified with 8 g L^−1^ bacto agar prior to autoclaving supplemented with spectinomycin thereafter.

In *barley* BCCM (Table 1) was completely removed and 600 *μ*L *A. tumefaciens* culture was added per well. The plate was placed in an exsiccator and vacuum infiltrated for 1 minute at 500 mbar. Then it was kept for 10 minutes inside the laminar hood without agitation followed by a washing step using 2.5 mL of BCCM. For co-cultivation the embryos were left in 2.5 mL of BCCM per well and the plates were incubated at 21°C in the dark for 48–72 hours without agitation.

In *wheat* 50 precultivated IEs were collected into one well of a 6-well plate and treated with 2.5 mL liquid PTM (Table 1) for 2 to 4 hours at RT. After removal of PTM 600 *μ*L *A. tumefaciens* culture was added, and the plate kept for 30 minutes inside the laminar hood. After washing twice with 2.5 mL WCCM (Table 1) IEs were placed in two stacks of 25 IEs each in a small petri dishes (ø 5.5 cm) on 4.5-cm sterile filter paper disks soaked with 400 *μ*L WCCM containing 100 mg L^−1^ Larcoll and incubated at 21°C in the dark for 48–72 hours (see Note 10).

In *triticale* 25 precultivated IEs were transferred into liquid BCCM (Table 1) right prior to co-cultivation (see Note 11). The following steps were conducted as described for *barley* except that the washed IEs were placed in stacks onto filter paper disks soaked with 300 *μ*L of BCCM as described for *wheat*.

For *maize* transformation *A. tumefaciens* was precultivated for 2-3 d on solid CPY with 100 mg L^−1^ spectinomycin at 21°C in the dark. On the day of transformation the *Agrobacterium* colonies were collected from the plate with a spatula, resuspended in IM (Table 1) and incubated 2-3 hours at 23°C and 100 rpm. OD_600_ was adjusted to 0.7. For inoculation the collected IEs were washed once with 1 mL IM. Then 1 mL of *Agrobacterium* suspension was added and mixed by inverting the tube. After incubation of 5 minutes at room temperature the IEs were transferred to four dry 4.5-cm filter paper disks to remove excess solution. Subsequently 40 IEs each were placed with the scutellum side up onto petri dishes containing MCCM (Table 1).

### 3.3. Callus Development, Regeneration, and Rooting

In *barley* 10 IEs were cultivated per petri dish containing BCIM (Table 1) (see Note 12). The IEs were placed onto the medium with the scutellum side facing down. Sealed petri dishes were incubated in the dark at 24°C for two weeks followed by a subcultivation on fresh medium for another two weeks. 

In *wheat* 25 IEs per petri dish were cultivated containing WCIM (Table 1) and incubated at 24°C in the dark for 10 d the scutellum facing upwards. Next the IEs were incubated for another week on WCIM containing 20 mg L^−1^ hygromycin under the same conditions (see Note 13).

After co-cultivation, *triticale* embryos 10 each were transferred to petri dishes containing solid BCIM (Table 1) and cultivated for 2 weeks followed by subcultivation on fresh medium additionally supplemented with 25 mg L^−1^ hygromycin for another 2 weeks (see Note 14).

In *maize* 40 IEs were incubated first on MCIM (Table 1) at 24°C in the dark for 7 d. For the first selection of two weeks they were transferred to MCIM containing 1.5 mg L^−1^ bialaphos (Molekula, Germany). In the second selection step 20 embryos were cultivated per dish on MCIM supplemented with 3 mg L^−1^ bialaphos. The medium was replaced every 14 d for up to three months until white, rapidly growing type II calluses emerged (see Note 15). 

Four weeks after gene transfer, the *barley* and *triticale* calluses were plated onto BRM (Table 1) (see Note 16). The plates were incubated at 24°C under illumination at 136 *μ*mol s^−1^ m^−2^ photon flux density for 16 hours per day. BRM was replaced fortnightly until regenerants emerged. Plantlets with a leaf length of 2 to 3 cm were then individually grown in glass tubes (100 mm, ø 25 mm; Schütt, Germany) containing 4.5 mL of BRM (see Note 17). Rooted plants were transferred to the glasshouse where they grew to maturity under the same conditions as described for the donor plants.

The regeneration step for *wheat* was performed with 10 embryo-derived calluses per petri dish containing BRM (Table 1) (see Note 18) supplemented with 25 mg L^−1^ hygromycin for two weeks at 136 *μ*mol s^−1^ m^−2^ photon flux density for 16 hours per day at 22°C. The calluses showing green tissue were selected and again transferred to BRM and incubated under identical conditions for another 2x 14 d until shoot formation. Plantlets with a leaf length of 2 to 3 cm were treated like *barley* plants.

Segments of *maize* calluses with immature somatic embryos were placed first on MRM (Table 1) with 1.5 mg L^−1^ bialaphos for one week in the dark followed by another week incubation in the light with 16 hours photoperiod of 170 *μ*mol s^−1^ m^−2^ photon flux density at 24°C. Matured somatic embryos were removed from the callus under a preparation microscope and incubated in high petri dishes (100 × 20 mm, Greiner, Germany) containing MRM supplemented with 1.5 mg L^−1^ bialaphos for a further two weeks until plantlets were formed. These plantlets were grown in culture vessels (107 × 94 × 96, SteriVent high, Duchefa, The Netherlands) with MRM (Table 1) for up to 14 d until they reached a size of approximately 10 cm. Then they were potted into soil (Substrat 2, Klasmann, Germany) and cultivated as described for the donor plants.

### 3.4. Analysis of Transgenic Material

**Figure 1:**
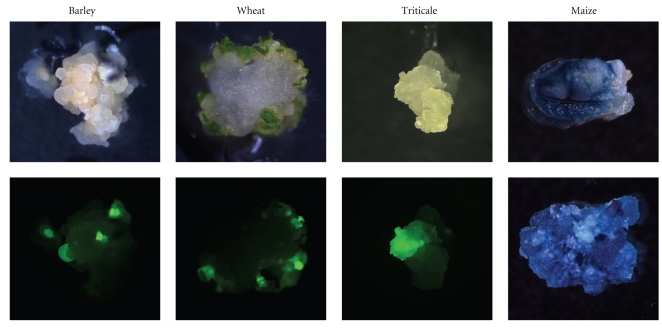
Reporter gene expression in immature embryo-derived calluses for three weeks (barley), four weeks (wheat), two weeks (triticale), 12 d (maize, upper picture) and 8 weeks (maize, lower picture) after co-culture. On the lower pictures of barley, wheat and triticale, the same objects are shown as above, but being exposed to far blue light and recorded with a GFP-filter set. The calluses of maize are shown following histochemical GUS assay [41].

**Figure 2:**
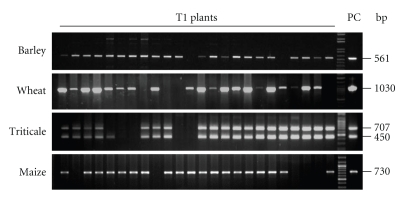
PCR analysis of progenies of primary transgenic plants. Twenty four plants of each T1 family were analysed for the presence of s*gfp* (barley, wheat, triticale, lower bands), *Hpt* (triticale, upper bands) or *Gus* (maize).

In order to facilitate the evaluation of the gene transfer and regeneration process, reporter genes were used instead of effector genes during the period of method establishment (Figure 1). For PCR analysis, genomic DNA from approximately 100 mg of leaf material stored in liquid nitrogen was isolated by means of commercially available extraction kits (e.g., DNAzol, Invitrogen, Germany) according to the manufacturer's instructions. Standard PCR reactions with the appropriate primers (Table 2) were performed using 100 ng genomic DNA per candidate plant. The PCR products were visualised following gel electrophoresis (Figure 2). 

Plants which had proven PCR-positive were further analysed by Southern blot for transgene integration and copy number (data not shown). To this end, high quality DNA was prepared as described by Pallotta et al. [42]. Twenty five *μ*g genomic DNA was digested with the appropriate restriction enzyme and the obtained fragments were separated by gel electrophoresis and blotted onto a hybond N membrane (Roche, Germany). Hybridisation of the blotted DNA with a gene-specific probe was done by labelling with DIG following the manufacturer's instructions (Roche, Germany).

**Notes**

(1) The substrate mix is a special white peat substrate plus clay to ensure adequate pH buffering.

(2) Osmocote is a general long-term fertilizer that contains 19% N, 6% P and 12% K. Hakaphos Blau is a general fertilizer that contains 15% N, 10% P and 15% K. Hakaphos Grün is a general fertilizer that contains 20% N, 5% P and 10% K.

(3) Substrate 2 consists of black and white peat. After germination plants are transferred to a substrate mix (compost, sand and white peat).

(4) Osmocote Pro is a general long-term fertilizer that contains 19% N, 7% P and 10% K.

(5) Notably, the developmental stage of the IEs is more crucial than their size. For the protocols described here, transition stage IEs that are about to turn from translucent to white colour are suited best.

(6) Contradictory results have been published regarding the effect of Acetosyringone on *Agrobacterium*-mediated transformation of immature barley embryos [9, 43, 44]. The addition of Acetosyringone results in increased transformation efficiency under the conditions described here.

(7) L-Cysteine supplemented to the co-culture medium was reported to prevent embryos from browning upon inoculation with *Agrobacterium* and to increase the transformation efficiency in soybean [45].

(8) In general, there is a risk to drop a plasmid when *Agrobacterium* is grown in the absence of antibiotics. However, in the protocol described here there was not any loss of vector detected, although *Agrobacterium* used for transformation was repeatedly checked via plasmid preparation. The advantage of growing *Agrobacterium* without antibiotics prior to inoculation is that the grown suspension can be directly used and the recipient cells are not exposed to any residual antibiotics.

(9) In barley, co-cultivation in liquid medium permits a substantially increased number of immature embryos to be processed at once, which results in a remarkable improvement in terms of efficiency [18].

(10) According to our experience, wheat IEs do not tolerate co-culture in liquid medium. On the other hand, it was shown earlier that wheat transformation efficiency can be improved through slight desiccation of IEs [3]. In the protocol presented here, gene transfer to wheat IEs is conducted on filter discs soaked with co-culture medium.

(11) Triticale IEs do not tolerate liquid co-culture as is the case in wheat.

(12) The increased CuSO_4_ concentration [46] results in improved formation of green plants compared to the conditions described by Tingay et al. [9].

(13) In wheat a resting period without selection following co-culture turned out to be crucial for the generation of transgenic lines.

(14) Although a comparatively low hygromycin concentration was used for cv. “Bogo”, all regenerants obtained proved transgenic.

(15) Depending on the genotype different callus types are recommended for manual selection [17].

(16) FHG medium has been successfully used for plant regeneration in a number of published experiments [7, 9, 44, 47]. Yet, a direct comparison conducted in our lab revealed that BRM (Table 1) is superior to FHG.

(17) Alternatively, as many as 16 plants can be grown per culture vessels (see maize) containing BRM. However, glass tubes are preferred to minimize the risk of cross contamination.

(18) Several media have been described for the selective development of transgenic wheat regenerants [3]. In our experiments selection worked best on BRM supplemented with hygromycin (Table 1).

## 4. Conclusion

**Figure 3:**
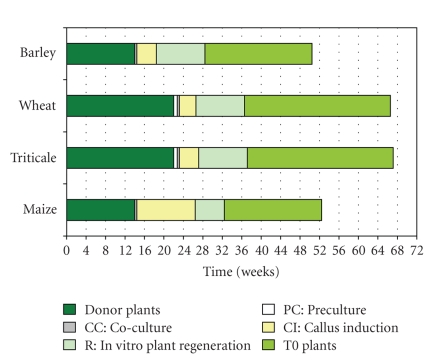
Time lines covering the entire transformation process from growing the donor plants until mature grains can be harvested from primary transgenic plants.

In this paper, effective and reproducible protocols for the generation of stably transgenic barley, wheat, triticale and maize plants are presented. In comparison with the earlier reports several improvements have been implemented. The selection regimes utilized for all four species proved to ensure an almost exclusive regeneration of transgenic plants, which is valid for both hygromycin-based selection in barley, wheat and triticale as well as selection of transgenic maize which relies on bialaphos. The period of time needed for the entire process from growing donor plants until the harvest of mature grains from primary transgenic lines is between 51 weeks in spring barley and maize up to 66 weeks in winter wheat and triticale (Figure 3). The transformation efficiencies obtained by the methods described have been 20–86% in barley, 2–10% in wheat, 2–4% in triticale and 0.5–24% in maize. The presented protocols are suitable for comprehensive functional analyses of recombinant nucleotide sequences on a large scale. Furthermore, they constitute a powerful fundament for applied research aiming to improve, for example, disease resistance, tolerance towards abiotic stresses as well as product quality of cereal crops.

## Figures and Tables

**Table 1:** Details on the transformation procedures and the materials needed in barley, wheat, triticale and maize. MS (Murashige and Skoog, for example, Duchefa no. M0221), K4N [11], B5 (Gamborg B5 Vitamin Mixture, e.g., Duchefa no. G0415), Hygromycin (Hygromycin B, e.g., Roche no. 10843555001), IEs—immature embryos. In cases where it is necessary to distinguish different medium compositions, the generic abbreviations of media (PCM, CCM, CIM and RM) are preceeded by a capital letter (B for barley, W for wheat, T for triticale and M for maize) representing the species for which a particular medium has been initially developed.

Treatment/Step	Barley	Wheat	Triticale	Maize
Embryo precultivation	—	Scutellum directed up, 5 d on WPCM (4.3 gL^−1^ MS minerals, 5 *μ*M CuSO_4_, 103.1 mgL^−1 ^MS vitamins, 0.5 gL^−1^ Glutamine, 8 mgL^−1 ^Dicamba, 40 gL^−1^ Maltose·H_2_O, 0.1 gL^−1^ Casein hydrolysate, pH = 5.8, 2.5 gL^−1^ Phytagel), 24°C, dark. Incubate 50 IEs per well for 2–4 hours in 6-well plate with 2.5 mL PTM (4.3 gL^−1^ MS minerals, 5 *μ*M CuSO_4_, 103.1 mgL^−1^ MS vitamins, 0.5 gL^−1^ Glutamine, 2 mgL^−1^ 2,4-D, 63.75 gL^−1^ Mannitol-D, 40 gL^−1^ Maltose·H_2_O, 0.1 gL^−1^ Casein hydrolysate, pH = 5.8), RT, dark	Scutellum directed up, 5 d on TPCM (4.3 gL^−1^ MS minerals, 103.1 mgL^−1^ MS vitamins, 0.5 gL^−1^ Glutamine, 6.6 mgL^−1^ Dicamba, 15 gL^−1^ Glucose, 15 gL^−1^ Sucrose, 200 *μ*M Acetosyringone, 0.1 gL^−1^ Casein hydrolysate, pH = 5.2, 2.5 gL^−1^ Phytagel), 24°C, dark	—

Inoculation	30–50 IEs in a 6-well plate with 2.5 mL BCCM (4.3 gL^−1^ MS minerals, 1 mgL^−1^ Thiamine HCl, 0.8 gL^−1^ L-Cysteine, 0.69 gL^−1^ L-Proline, 2.5 mgL^−1^ Dicamba, 30 gL^−1^ Maltose·H_2_O, 500 *μ*M Acetosyringone, 1 gL^−1^ Casein hydrolysate, 0.25 gL^−1^ Myo-inositol, pH = 5.8) each. Remove BCCM and add 600 *μ*L *Agrobacterium* OD_600_ = 2–2.5, 1 minute 500 mbar, 10 minutes resting at RT, wash for 15 minutes, BCCM	Remove PTM and add 400 *μ*L *Agrobacterium*, OD_600 _ = 2–2.5, 30 minutes resting at RT, wash 2x for 5 minutes, WCCM (4.3 gL^−1^ MS minerals, 103.1 mgL^−1^ MS vitamins, 0.8 gL^−1^ L-Cysteine, 0.5 gL^−1^ Glutamine, 6 mgL^−1 ^2,4-D, 15 gL^−1^ Glucose, 15 gL^−1^ Sucrose, 500 *μ*M Acetosyringone, 0.1 gL^−1^ Casein hydrolysate, pH = 5.8)	Collect 25 precultivated IEs to 2.5 mL BCCM (see barley for media composition). Remove BCCM and add 600 *μ*L^−1^ *Agrobacterium* OD_600_ = 2.5–3, 1 minute 500 mbar, 10 minutes resting at RT, wash 1-2x for 5 minute, BCCM (see barley for media composition)	Collect up to 200 IEs in 1 mL IM (4 gL^−1^ Chu N6 salt mixture, 4 mgL^−1 ^Chu N6 vitamins, 0.7 gL^−1^ L-Proline, 1.5 mgL^−1^ 2,4-D, 36 gL^−1^ Glucose, 68.4 gL^−1^ Sucrose, 100 *μ*M Acetosyringone, pH = 5.2), wash 1x, remove IM, add 1ml IM with *Agrobacterium* OD_600_ = 0.7, 5 minutes resting at RT, blot IEs dry on 4 filter papers (ø 4.5 cm)

Co-cultivation	48–72 hours in 2.5 mL BCCM (see inoculation for composition), 21°C, dark	48–72 hours, 25 IEs as stack on filter paper (ø 4.5 cm) soaked with 400 *μ*L WCCM (see inoculation for composition) + 100 mgL^−1 ^Larcoll, in petri dish (ø 5.5 cm), 21°C, dark	48–72 hours, 25 IEs as stack on filter paper (ø 4.5 cm) soaked with 300 *μ*L BCCM (see barley for composition), in petri dish (ø 5.5 cm), 21°C, dark	48–72 hours, 40 IEs on MCCM (2 gL^−1^ Chu N6 salt mixture, 2 mM CaCl_2_, 112 mgL^−1^ B5 vitamins, 0.4 gL^−1^ L-Cysteine, 2.9 gL^−1^ L-Proline, 4.4 mgL^−1^ Dicamba, 37.6 gL^−1^ Maltose·H_2_O, 100 *μ*M Acetosyringone, 1 mM DTT, 0.5 gL^−1^ MES, pH = 5.8, 4 gL^−1^ Phytagel), 21°C, dark

Callus induction	10 IEs each for 2x 14 d on BCIM (4.3 gL^−1^ MS minerals, 5 *μ*M CuSO_4_, 1 mgL^−1^ Thiamine HCl, 0.69 gL^−1^ L-Proline, 2.5 mgL^−1^ Dicamba, 30 gL^−1^ Maltose·H_2_O, 1 gL^−1^ Casein hydrolysate, 0.25 gL^−1^ Myo-inositol, pH = 5.8, 3 gL^−1^ Phytagel, 150 mgL^−1^ Timentin) + 50 mgL^−1^ Hygromycin, 24°C, dark	25 IEs each for 10 d on WCIM (4.3 gL^−1^ MS minerals, 5 *μ*M CuSO_4_, 103.1 mgL^−1^ MS vitamins, 0.5 gL^−1^ Glutamine, 2 mgL^−1 ^2,4-D, 40 gL^−1^ Maltose *·*H_2_O, 0.1 gL^−1^ Casein hydrolysate, pH = 5.8, 3 gL^−1^ Phytagel, 150 mgL^−1^ Timentin), 24°C, dark, 25 IEs each for 7 d on WCIM + 20 mgL^−1^ Hygromycin, 24°C, dark	10 IEs each for 14 d on BCIM (see barley for composition) without Hygromycin, 24°C, dark, 14 d on BCIM + 25 mgL^−1^ Hygromycin, 24°C, dark	40 IEs each for 7 d on MCIM (4 gL^−1^ Chu N6 salt mixture, 2 mM CaCl_2_, 5 *μ*M silver nitrate, 112 mgL^−1^ B5 vitamins, 2.9 gL^−1^ L-Proline, 4.4 mgL^−1^ Dicamba, 34.2 gL^−1^ Sucrose, 0.1 gL^−1^ Casein hydrolysate, 0.5 gL^−1^ MES, pH = 5.8, 4 gL^−1^ Phytagel, 150 mgL^−1^ Timentin), 20 IEs each for 14 d on MCIM + 1.5 mgL^−1^ Bialaphos, 4–7x 14 d on MCIM + 3 mgL^−1^ Bialaphos, 24°C, dark

Shoot formation	3x 14 d on BRM (K4N minerals, 112 mgL^−1^ B5 vitamins, 146 mgL^−1 ^L-Glutamine, 0.225 mgL^−1 ^6-BAP, 36gL^−1 ^Maltose·H_2_O, pH = 5.8, 3 gL^−1 ^Phytagel, 150 mgL^−1^ Timentin) + 25 mgL^−1^ Hygromycin, 24°C, 16 hours light (136 *μ*mol s^−1^ m^−2^)	see barley	see barley	6–10 calluses for 7 d on MRM (4.3 gL^−1^ MS minerals, 2 mM CaCl_2_, 103.1 mgL^−1^ MS vitamins, 60 gL^−1 ^Sucrose, 0.1 gL^−1 ^Myo-inositol, pH = 5.8, 3 gL^−1 ^Phytagel, 75 mgL^−1 ^Timentin) + 1.5 mgL^−1^ Bialaphos, 24°C, dark, 2x 14 d on MRM + 1.5 mgL^−1 ^Bialaphos, in high petri dishes (100 × 20 mm), 24°C, 16 hours light (170 *μ*mol s^−1^ m^−2^)

Plantlet formation	Each plant for 14–28 d on BRM + 25 mgL^−1^ Hygromycin, in culture vessels (see maize), 24°C, 16 hours light (136 *μ*mol s^−1^ m^−2^)	see barley	see barley	6 plants for 7–14 d on MRM (half strength sucrose compared to shoot formation), in culture vessels (107 × 94 × 96 mm), 24°C, 16 hours light (170 *μ*mol s^−1^ m^−2^)

Plant establishment in soil	5-6 weeks in substrate mix (Spezialmischung Petuniensubstrat, Klasmann, Germany), 40g fertiliser “Osmocote” (Scotts, Netherlands) per 7.5 L pot, 14/12°C day/night, 12 hours light (136 *μ*mol s^−1^ m^−2^)	see barley	see barley	2–4 weeks in “Substrat 2” (Klasmann, Germany), 22/20°C day/night, 16 hours light (170 *μ*mol s^−1^ m^−2^)

**Table 2:** PCR-Primer used for the analysis of transgenic plants.

Primer	Sequence 5′–3′
GH-Hpt-F1	GAT CGG ACG ATT GCG TCG CA
GH-Hpt-R2	TAT CGG CAC TTT GCA TCG GC
GH-Gfp-F1	GGT CAC GAA CTC CAG CAG GA
GH-Gfp-R1	GAC CAC ATG AAG CAG CAC GA
GH-Gfp-R2	TAC GGC AAG CTG ACC CTG AA
GH-Gus-F1	CCG GTT CGT TGG CAA TAC TC
GH-Gus-R1	CGC AGC GTA ATG CTC TAC AC
GH-Ubi-F1	TTC CGC AGA CGG GAT CGA TCT AGG
